# Determinants of adverse event occurrence in children with short stature born small for gestational age treated with growth hormone

**DOI:** 10.3389/fendo.2025.1656966

**Published:** 2025-11-19

**Authors:** Regis Coutant, Nazim Benchikh, Agnès Linglart, Marc Nicolino, Jean-Pierre Salles

**Affiliations:** 1 Department of Paediatric Endocrinology, University Hospital, Angers, France; 2 Rare Endocrine Disorders, Clinical Medical and Regulatory, Novo Nordisk, Paris, France; 3 AP-HP, Paris Saclay University, INSERM, Physiologie et Physiopathologie Endocriniennes, Endocrinology and Diabetology for Children, Bicêtre Paris Saclay Hospital, Le Kremlin-Bicetre, France; 4 Division of Pediatric Endocrinology and Metabolism, Lyon University Pediatric Hospital, Lyon, France; 5 Endocrinology and Bone Diseases Unit, Children’s Hospital, Toulouse University Hospital, University of Toulouse Paul Sabatier, INSERM-CNRS Unit 1291, Infinity Center, Toulouse, France

**Keywords:** growth hormone, small for gestational age, adverse events, serious adverse events, real-world data

## Abstract

**Introduction:**

Recombinant human growth hormone (rhGH) treatment for short children born small for gestational age (SGA) is effective in improving height outcomes. Determinants of height response to rhGH treatment have been identified using multiple regression analyses. Although adverse events (AEs) associated with treatment have been described, determinants of AE occurrence are not well known.

**Methods:**

This analysis used safety data collected between 2007 and 2018 in an observational, prospective registry study (NCT01578135) including children born SGA across 126 sites in France. AEs were reported by patients or recorded by treating physicians. Univariate and multivariate analyses were performed to identify determinants of occurrence of AEs and serious AEs (SAEs).

**Results:**

Of 291 patients from the registry, 287 AEs were reported in 149 (51.2%) patients. Each patient experienced a mean of 0.205 AEs per patient-year (range 0–4.3) and a mean of 0.054 SAEs (range 0–1.8). The most frequently reported AEs were headache (9.3%) and arthralgia (4.5%). Using multiple regression analyses, longer registry participation (p=0.0146, odds ratio [OR]=1.98), presence of chronic disease (p=0.0004, OR = 2.56), and concomitant treatment (p=0.0162, OR = 1.79) were associated with increased risk of experiencing AEs. GH dose at inclusion or cumulative GH dose during first 2 years of treatment were not associated with AE risk, while higher GH dose at last visit was associated with reduced risk (p=0.0412, OR = 0.58).

**Conclusions:**

In short children born SGA, underlying conditions and associated treatments seem to be the main factors associated with AE occurrence, while GH dose was inversely related to AE occurrence.

## 1 Introduction

Most children born small for gestational age (SGA), characterized by low birth weight and/or length below −2 standard deviation scores (SDS) relative to the reference population, typically exhibit catch-up growth during their early years (1–3). However, roughly 10−15% of children born SGA do not exhibit catch-up growth and instead experience persistent short stature into later childhood and short final height (3, 4). Current consensus guidelines recommend growth hormone (GH) treatment for children born SGA with short stature when rapid catch-up growth is no longer likely (usually by age 3–4 years), unless another cause of short stature can be determined (5). Consequently, short stature in children born SGA is the second most common indication for GH treatment after GH deficiency (GHD); GH treatment in short children born SGA is authorized from the age of 4 years by the European Medicines Agency (5, 6).

Several studies have reported final height response to recombinant human GH (rhGH) in treated children with SGA (7–15), as well as the determinants of response using regression analyses (13, 16–18). Cause of SGA, birth length SDS, duration of treatment, height at start of GH treatment, GH dose, and midparental height were among the most frequently identified determinants of response. These studies have also shown that the safety profile of GH treatment, at recommended doses for children born SGA, is good with low overall incidence of serious adverse events (SAEs) (16–18). Nevertheless, reports on the long-term safety of GH treatment in patients with short stature born SGA, as well as in patients with other causes of GH-treated short stature, are limited.

While published data from the Safety and Appropriateness of Growth hormone treatments in Europe (SAGhE) project indicate that GH treatment is not associated with an increased risk of cancer incidence or mortality, risk of circulatory and cerebrovascular disease development appears to be raised in short children born SGA treated with GH (19–21). However, it is unclear whether this increased risk is attributable to GH treatment specifically or to other factors, such as the disease causing the prenatal growth retardation (20, 21). Therefore, ongoing characterization of the safety profile of GH treatment remains relevant. In this regard, an increased understanding of the determinants associated with the occurrence of adverse events (AEs) during GH therapy may be helpful in informing clinical practice and optimizing outcomes for patients.

In France, a national registry was created in 2006 to address the data gap on the effectiveness and tolerability of long-term GH treatment with Norditropin SimpleXx^®^ (Novo Nordisk A/S, Bagsværd, Denmark) in children born SGA (16). This registry was requested by the French health authority to provide follow-up data and was associated with a systematic report of the growth response to treatment and AEs (16). The primary endpoint of the initial registry analysis, which has been published previously, was the proportion of patients achieving normalized height SDS at last visit (i.e., >−2 relative to the reference population) (16). Around two-thirds of patients (66.3%; n=193) achieved normalized height SDS (>−2 relative to the reference population) at the last visit. A total of 24.7% (n=72) of patients reached final adult height (FAH). Of these, 55.6% (n=40) reached FAH SDS >−2 when calculated relative to adult age and not to chronological age (16). Among children who reached final adult height, mean (SD) height SDS at the last visit was -1.8 (0.9) and -2.0 (0.84) relative to chronological age and adult age, respectively.

Here, we report real-world safety data from French children born SGA treated with Norditropin SimpleXx^®^ who were enrolled in this registry. More specifically, we investigate the potential determinants associated with the onset and number of serious and nonserious AEs in these patients.

## 2 Materials and methods

### 2.1 Study design, patients, and treatment

This observational, multicenter, noninterventional study (ClinicalTrials.gov NCT01578135) was conducted to assess the effectiveness and safety of daily GH treatment in French children born SGA across 126 sites in France. The study design, population, and treatment have been previously described (16). Patient data were prospectively collected from 18 March 2007 to 9 October 2018, and retrospectively collected between 29 April 2005 and 17 March 2007. The last patient was included in the database on 29 April 2010. Hence, the study inclusion period was 5 years in total (2005–2010). Of all enrolled patients, a representative subpopulation of every one in five patients (in the order of appearance in the centralized patient register) were systematically selected for prospective follow-up. The first follow-up visit was 6 months after inclusion and then visits occurred annually until achieving FAH (defined as height velocity <2 cm/year; or bone age >14 years for girls and >16 years for boys) or the study termination, whichever occurred first.

The study population included any patient with short stature born SGA treated with daily GH for growth retardation, regardless of whether they were treatment-naïve (this was an initial GH prescription) or if they were previously treated with GH (had previously received GH at least one day before study inclusion). Daily GH (Norditropin^®^, Novo Nordisk A/S, Bagsværd, Denmark) was prescribed for subcutaneous injection as ready-to-use cartridges in an injection pen (NordiPen^®^, Novo Nordisk A/S, Bagsværd, Denmark). Daily GH was prescribed in a hospital setting on an Exception Drug Status medication prescription by a pediatric hospital practitioner or endocrinology and metabolic disorders specialist. As in routine practice, and according to local regulations, packaged and labelled study product was available by prescription and by purchase or supply. In this observational study, the sponsor did not supply patients with GH. As treatment decisions were made at the physician’s discretion, in line with usual clinical practice, the duration of GH treatment was not specified in the study protocol and patients were to be treated until achieving adult height.

This study was conducted in accordance with the Declaration of Helsinki (22, 23), Guidelines for Good Pharmacoepidemiology Practice (24), and regulatory requirements. Informed written consent was granted by the parent(s)/or a legally authorized representatives before any study-related activities for all patients aged under 18 years, and patients could withdraw from the study at will at any time. The patient could also be withdrawn from the study at the discretion of the physician or sponsor due to a safety concern.

### 2.2 Safety assessments

Due to concerns raised by the French health authorities regarding the safety of GH treatment, particular attention was paid to the reporting of AEs. AEs were reported by the patients or their treating physician and were classified before database lock according to Medical Dictionary for Regulatory Activities (MedDRA) terminology. Any AE was considered serious if it met one of the following standard severity criteria: death; life-threatening event; hospital admission or extended hospital stay; permanent or significant disability; congenital anomaly or neonatal malformation; or a significant medical event. The investigator was to forward information relating to all SAEs to the trial conductors within 24 hours of becoming aware of the event.

The relatedness of AEs and SAEs to GH treatment was determined by the patient’s physician. This relationship reflected only the opinion of the physician and did not account for GH dose, treatment duration, the decision to stop treatment, or the effect of stopping treatment. In addition, it was not validated by an independent committee. Events related to tumor, cardiovascular, and cerebrovascular pathologies were identified as being of particular interest for descriptive analysis.

### 2.3 Analysis of determinants associated with reported AEs and SAEs

The following potential determinants were identified for investigation: age at treatment start, auxological characteristics, GH dose (mean and cumulative), GH treatment duration, puberty status, bone maturation, chronic diseases, and concomitant medications. Puberty was defined as Tanner Stage S2 for girls and mean volume of both testicles ≥4 mL for boys, and bone age was assessed locally by the treating physician using the Greulich-Pyle method (25). Chronic diseases were assessed at treatment initiation and were classified before database freeze according to MedDRA terminology. Concomitant medications were classified by therapeutic field according to the World Health Organization drug dictionary (Anatomical Therapeutic Chemical classification system).

### 2.4 Statistical analyses

Statistical analyses were performed using SAS 9.4 software. Baseline data and safety outcomes were summarized with descriptive statistics for all patients and for patient subgroups divided by GH treatment status at baseline. The analysis of determinants associated with reported AEs and SAEs was performed by univariate analysis using a binary logistic regression model on each separate pre-selected parameter. These regression analyses were performed to specifically assess factors associated with the occurrence of ≥1 AE, the occurrence of ≥1 SAE, or the number of AEs or SAEs. Factors with a <20% significance level in the univariate analysis were then included in the subsequent analysis using a multivariable logistic regression model with stepwise backward selection to remove nonsignificant variables (p≥0.05). The p-values were calculated for the overall association between the parameter and the dependent variables described above (occurrence of ≥1 AE/SAE or the number of AEs/SAEs). Determinants were considered to be associated with an increased risk of AEs or SAEs if the odds ratio (OR) was >1 and the p-value was <0.05.

## 3 Results

### 3.1 Baseline characteristics

In total, 1408 patients were included in the register. Two children were excluded due to a reported diagnosis of Turner syndrome, leaving 1406 patients. From these patients, one in every fifth child was randomly selected, providing 291 patients for inclusion in the long-term follow-up study. Mean (standard deviation [SD]) age at inclusion of patients undergoing long-term follow-up was 8.08 (3.32) years (**Table 1**).

**Table 1:** Baseline characteristics of the follow-up population by GH treatment status at inclusion.

Variable	Previously treated (n=109)	Treatment-naïve (n=182)	Total (n=291)
Sex, n (%):			
Male	62 (56.9)	95 (52.2)	157 (54.0)
Female	47 (43.1)	87 (47.8)	134 (46.0)
Age at inclusion in years, mean (SD)	8.48 (3.20)	7.85 (3.38)	8.08 (3.32)
Age at treatment initiation in years, mean (SD)	5.35 (2.84)	7.85 (3.38)	6.93 (3.40)
Gestational age (weeks), mean (SD)	36.37 (4.34)	37.30 (3.57)	36.96 (3.89)
Puberty onset at inclusion, n (%)	20 (18.3)	17 (9.3)	37 (12.7)
Bone maturation at inclusion* (years), mean (SD)	1.10 (1.38)	1.71 (1.35)	1.50 (1.38)
Birth length SDS for gestational age, mean (SD)	–3.15 (1.44)	–2.59 (1.09)	−2.79 (1.25)
Birth weight SDS for gestational age, mean (SD)	–2.18 (1.07)	–1.66 (1.03)	−1.85 (1.07)
Weight for CA at treatment initiation, mean (SD)	–2.64 (1.04)	–2.09 (0.88)	–2.29 (0.97)
Height for CA at initiation (cm)	92.41 (11.29)	99.98 (14.33)	95.91 (13.10)
Height SDS for CA at treatment initiation, mean (SD)	–3.25 (1.00)	–2.97 (0.76)	−3.07 (0.86)
Height velocity SDS at treatment initiation (SDS/year), mean (SD)	–1.05 (1.63)	–1.29 (1.89)	−1.18 (1.77)
Mother height SDS, mean (SD)	–1.15 (1.35)	–0.99 (1.18)	–1.05 (1.25)
Father height SDS, mean (SD)	–0.84 (1.09)	–0.90 (1.14)	–0.88 (1.12)
Target height SDS (SD)	–0.98 (0.93)	–0.94 (0.89)	−0.96 (0.91)
IGF-I SDS at inclusion, mean (SD)	1.23 (2.03)	−0.91 (1.62)	0.40 (2.15)
Proportions of patients with IGF-I SDS, level:			
<−2, n (%)	4 (6.5)	8 (20.5)	12 (11.9)
−2 ≥ n ≤ +2, n (%)	35 (56.5)	30 (76.9)	65 (64.4)
>+2, n (%)	23 (37.1)	1 (2.6)	24 (23.8)
GH dose at inclusion (mg/kg/day), mean (SD)	0.0535 (0.0544)	0.0413 (0.0104)	0.0458 (0.0347)
Proportion of patients with GH dose at inclusion (mg/kg/day), range:			
<0.032, n (%)	2 (2.0)	17 (9.9)	19 (7.0)
0.032 ≥ n ≤0.038, n (%)	31 (30.4)	77 (45.0)	108 (39.6)
>0.038, n (%)	69 (67.6)	77 (45.0)	146 (53.5)
Treatment duration before study (years), mean (SD)	2.68 (1.80)	N/A	2.68 (1.80)
Treatment duration during study (years), mean (SD)	5.50 (3.26)	5.49 (2.85)	5.49 (2.98)
Presence of a chronic disease, n (%)	65 (59.6)	96 (52.8)	161 (55.3)
Receiving concomitant medication, n (%)	44 (40.37)	73 (40.11)	117 (40.21)

*Difference between chronological age and bone age at inclusion.

CA, chronological age; GH, growth hormone; IGF-I, insulin-like growth factor-I; N/A, not applicable; SD, standard deviation; SDS, standard deviation score.

A total of 334 chronic diseases were reported in 161 (55.3%) patients. The proportion of previously treated patients with daily GH vs. treatment-naïve patients with at least one chronic disease was similar (n=65 [59.6%] vs. n=96 [52.8%], respectively). Among the most commonly recorded chronic diseases were asthma (n=16), GHD (n=14), and psychomotor hyperactivity (n=10). In total, 23.4% (n=68) of patients had at least one congenital, familial, or genetic disorder, the most common of which were dysmorphism (n=8), Silver-Russell syndrome (n=7), Noonan syndrome (n=6), and fetal alcohol syndrome (n=6).

Concomitant medication use was recorded in 40.2% (n=117) of patients (previously treated patients: n=44 [40.4%]; treatment-naïve patients: n=73 [40.1%]). The most frequently administered concomitant medications were the gonadotropin-releasing hormone (GnRH) analogues leuprorelin (n=22; 7.6%) and triptorelin (n=18; 6.2%), the selective beta-2 adrenoreceptor antagonist salbutamol (n=15; 5.2%), and the thyroid hormone levothyroxine (n=13; 4.5%). Other concomitant medications received by patients with the potential to affect growth during the study included centrally acting sympathomimetics (n=11; 3.8%) and glucocorticoids (n=6; 2.1%).

### 3.2 GH dose at inclusion and during long-term follow-up

At inclusion, 146 patients (53.5%) were receiving a GH dose of >0.038 mg/kg/day (**Table 1**). The mean (SD) GH dose at treatment start was 0.0458 (0.0347) mg/kg/day for the whole cohort (n=291). Mean (SD) cumulative GH dose (mg/kg) during the first 2 years of treatment from the time of initiation was 30.23 (11.95).

### 3.3 Overview of reported AEs and SAEs

A total of 287 AEs were reported in 149 (51.2%) patients. The majority of AEs were nonserious (70.0%, n=201) (**Table 2**, **Supplementary Table 1**). In total, each patient (100%, n=291) experienced a mean of 0.986 AEs (range 0–15) and 0.296 SAEs (range 0–13) throughout the study. Numbers of AEs and SAEs per patient-year were lower in previously treated than in naïve patients (AEs: 0.771 vs. 1.115; SAEs: 0.284 vs. 0.324). Considering 266 patients with both AE and duration of exposure to treatment data (91.4%), each patient experienced a mean of 0.205 AEs per patient-year of exposure to treatment during the study (range 0–4.3) and a mean of 0.054 SAEs by the same measure (range 0–1.8).

**Table 2:** Overview of nonserious AEs reported in ≥2 patients in the follow-up population, grouped by treatment status.

Parameter	Previously treated (n=109)		Treatment-naïve (n=182)		Total (n=291)	
	Number of patients (%)	Number of AEs (%)	Number of patients (%)	Number of AEs (%)	Number of patients (%)	Number of AEs (%)
Patients with ≥ 1 nonserious AE/total number of nonserious AEs	42 (38.53)	57 (100.0)	81 (44.51)	144 (100.0)	123 (42.27)	201 (100.0)
Investigations						
IGF-I increased	17 (15.60)	18 (31.58)	33 (18.13)	41 (28.47)	50 (17.18)	59 (29.35)
Nervous system disorders						
Headache	3 (2.75)	3 (5.26)	24 (13.19)	24 (16.67)	27 (9.28)	27 (13.43)
Musculoskeletal and connective tissue disorders						
Arthralgia	8 (7.34)	8 (14.0)	5 (2.75)	6 (4.17)	13 (4.47)	14 (6.97)
Pain in extremity	0	0	5 (2.75)	5 (3.47)	5 (1.72)	5 (2.49)
Back pain	0	0	2 (1.10)	2 (1.39)	2 (0.69)	2 (1.00)
Myalgia	2 (1.83)	2 (3.51)	0	0	2 (0.69)	2 (1.00)
Respiratory, thoracic, and mediastinal disorders						
Asthma	1 (0.92)	1 (1.75)	4 (2.20)	5 (3.47)	5 (1.72)	6 (2.99)
Gastrointestinal disorders						
Abdominal pain	1 (0.92)	1 (1.75)	2 (1.10)	2 (1.39)	3 (1.03)	3 (1.49)
General disorders and administrative conditions						
Injection-site hematoma	1 (0.92)	1 (1.75)	2 (1.10)	3 (2.08)	3 (1.03)	4 (1.99)
Injection-site atrophy	0	0	2 (1.10)	2 (1.39)	2 (0.69)	2 (1.00)
Injection-site hemorrhage	1 (0.92)	1 (1.75)	1 (0.55)	1 (0.69)	2 (0.69)	2 (1.00)
Psychiatric disorders						
Abnormal behavior	0	0	2 (1.10)	2 (1.39)	2 (0.69)	2 (1.00)
Aggression	0	0	2 (1.10)	2 (1.39)	2 (0.69)	2 (1.00)
Agitation	0	0	2 (1.10)	2 (1.39)	2 (0.69)	2 (1.00)

AE, adverse event; IGF-I, insulin-like growth factor-I.

By preferred term, the most common nonserious AEs were increased insulin-like growth factor-I (IGF-I), above 2 SDS (59 events in 50 [17.2%] patients), headache (27 events in 27 [9.3%] patients), and arthralgia (14 events in 13 [4.5%] patients). Increased IGF-I occurred in a similar proportion of treatment-naïve patients and previously treated patients (n=33 [18.1%] and n=17 [15.6%], respectively), while the proportion of patients experiencing headache was higher for treatment-naïve patients than previously treated patients (n=24 [13.2%] vs. n=3 [2.8%], respectively). Two treatment-naïve patients displayed three psychiatric disorder characteristics each of: abnormal behavior, aggression, and agitation.

Overall, 16 (5.5%) patients discontinued treatment due to AEs. The most common reason for discontinuation was increased IGF-I, occurring in two treatment-naïve and two previously treated patients.

There were 86 SAEs reported in 46 patients (**Table 3**, **Supplementary Table 2**). The most common SAEs were appendicectomy, due to appendicitis (four events in four patients) and gastroenteritis (four events in two patients). Three events of acute respiratory distress syndrome occurred in two patients.

**Table 3:** Overview of SAEs reported in ≥2 patients in the follow-up population, grouped by treatment status.

Parameter	Previously treated (n=109)		Treatment-naïve (n=182)		Total (n=291)	
	Number of patients (%)	Number of SAEs (%)	Number of patients (%)	Number of SAEs (%)	Number of patients (%)	Number of SAEs (%)
Patients with ≥ 1 SAE/total number of SAEs	17 (15.60)	27 (100)	29 (15.93)	59 (100)	46 (15.81)	86 (100)
Gastroenteritis	2 (1.83)	2 (7.41)	2 (1.10)	2 (3.39)	4 (1.37)	4 (4.65)
Acute respiratory distress syndrome	1 (0.92)	1 (3.70)	1 (0.55)	2 (3.39)	2 (0.69)	3 (3.49)
Appendicectomy	0	0	4 (2.20)	4 (6.78)	4 (1.37)	4 (4.65)
Asthma	0	0	3 (1.65)	3 (5.08)	3 (1.03)	3 (3.49)
Cryptorchism	1 (0.92)	1 (3.70)	1 (0.55)	1 (1.69)	2 (0.69)	2 (2.33)
Gastro-esophageal reflux disease	1 (0.92)	1 (3.70)	1 (0.55)	1 (1.69)	2 (0.69)	2 (2.33)

SAE, serious adverse event.

### 3.4 Relatedness of AEs to treatment

Relatedness to treatment was rated by the treating physician for all (86/86) SAEs and nearly all (286/287) reported AEs. Of the 286 AEs, 39.2% (n=112) were considered probably or possibly related to treatment (**Supplementary Table 3**). AEs were considered unlikely to be related to treatment in 45.1% (n=129) of cases. Most AEs considered probably or possibly related to treatment were light in severity (58.4% and 66.7%, for AEs probably related and AEs possibly related, respectively). The majority (84.9%; n=73) of reported SAEs were considered unlikely to be related to treatment. Three SAEs considered probably related to treatment were reported: testicular infarction, type 2 diabetes (each reported in previously treated patients), and asthma (in a treatment-naïve patient). Three SAEs, all occurring in a single treatment-naïve patient, were considered possibly related to treatment: two events of epiphysiolysis and one hip arthroplasty due to epiphysiolysis (**Supplementary Table 4**). Relatedness to treatment was impossible to specify for 15.7% (n=45) AEs and 8.1% (n=7) SAEs.

In treatment-naïve patients, one event of hyperinsulinemia was reported as probably related to treatment and one event of increased HbA1c was reported as possibly related to treatment.

### 3.5 Determinants associated with AEs and SAEs

Three determinants were identified in the univariate analysis as being associated with an increased risk of experiencing an AE (**Table 4**): longer registry participation while receiving treatment (p<0.0001, odds ratio [OR] =2.76), presence of chronic disease (p=0.0001, OR = 2.56), and use of concomitant treatment (p=0.0162, OR = 1.79). In the subsequent multivariate analysis with backward stepwise selection, the determinants significantly associated with an increased risk of experiencing an AE were longer registry participation while receiving treatment (p=0.0146, OR = 1.98) and presence of chronic disease (p=0.0004, OR = 2.56). GH dose at inclusion, or cumulative GH dose during the first 2 years of treatment, was not associated with an increased risk of AEs. Conversely, higher GH dose at last visit was significantly associated with a reduced risk of experiencing an AE in the univariate (p=0.0136, OR = 0.56) and multivariate analyses (p=0.0412, OR = 0.58).

**Table 4:** Investigated determinants associated with experiencing an AE or SAE (univariate and multivariate logistic regression analyses).

Parameter	Univariate analysis			Multivariate analysis		
	n used	OR [95% CI]	P-value	n used	OR [95% CI]	P-value
Factors associated with experiencing an AE						
Registry participation while receiving treatment (years)						
Median: >4.9405 vs. ≤4.9405	266	2.756 [1.68; 4.53]	<0.0001	263	1.980 [1.14; 3.43]	0.0146
Chronic diseases (yes/no)						
Yes vs. no	291	2.555 [1.59; 4.11]	0.0001	263	2.559 [1.52; 4.31]	0.0004
Puberty onset at inclusion (yes/no)						
Yes vs. no	291	0.356 [0.17; 0.75]	0.0067	263	0.433 [0.18; 1.04]	0.0605
GH dose at last visit (mg/kg/day)						
Median >0.0406 vs. ≤0.0406	288	0.555 [0.35; 0.89]	0.0136	263	0.578 [0.34; 0.98]	0.0412
Concomitant treatment (yes/no)						
Yes vs. no	291	1.791 [1.11; 2.88]	0.0162			
Height SDS at inclusion						
>−3 vs. ≤−3	277	0.678 [0.41; 1.11]	0.1250			
Chronological age at treatment initiation (years)						
Median: >5.9863 vs. ≤5.9863	288	0.556 [0.35; 0.89]	0.0136			
Height SDS at last visit						
Median: >−2.4627 vs. ≤−2.4627	291	1.101 [0.70; 1.74]	0.6805			
Height velocity at treatment initiation (SDS/year)						
Median: >−1.2089 vs. ≤−1.2089	149	0.868 [0.45; 1.66]	0.6692			
Bone age at treatment initiation (years)						
Median: >7 vs. ≤7	114	0.459 [0.22; 0.97]	0.0417			
GH dose at inclusion (mg/kg/day)						
Median: >0.0392 vs. ≤0.0392	273	1.488 [0.92; 2.40]	0.1023			
IGF-I SDS at inclusion (reference <-2 SDS)			0.2718					
>+2 SDS vs. <−2 SDS	101	1.667 [0.41; 6.77]	0.4749			
−2 SDS to +2 SDS vs. <−2SDS	101	0.757 [0.22; 2.60]	0.6579			
Birth height SDS						
Median: >−2.4528 vs. ≤−2.4528	269	0.753 [0.47; 1.22]	0.2465			
Birth weight SDS						
Median: >−1.7643 vs. ≤−1.7643	278	0.687 [0.43; 1.10]	0.1193			
Cumulative dose during first 2 years of treatment (mg/kg)			0.8076					
25.5675 ± 10% vs. > 28.12425	266	0.953 [0.53; 1.71]	0.8708			
<23.01075 vs. >28.12425	266	0.823 [0.46; 1.48]	0.5155			
Bone maturation at inclusion (years)						
Median: >1.5106 vs. ≤1.5106	114	0.810 [0.39; 1.69]	0.5741			
BMI at inclusion (kg/m^2^)						
Median: >14.8739 vs. ≤14.8739	276	0.944 [0.59; 1.51]	0.8097			
Factors associated with experiencing an SAE						
Concomitant treatment (yes/no)						
Yes vs. no	291	4.302 [2.18; 8.50]	<0.0001	277	3.962 [1.97; 7.97]	0.0001
Height SDS at inclusion						
>−3 vs. ≤−3	277	0.388 [0.20; 0.75]	0.0046	277	0.396 [0.20; 0.78]	0.0074
Chronic diseases (yes/no)						
Yes vs. no	291	4.010 [1.86; 8.66]	0.0004			
Height SDS at last visit						
Median: >−2.4627 vs. ≤−2.4627	291	0.480 [0.25; 0.92]	0.0283			
Chronological age at treatment initiation (years)						
Median: >5.9863 vs. ≤5.9863	288	0.496 [0.26; 0.96]	0.0372			
Height velocity at treatment initiation (SDS/year)						
Median: >−1.2089 vs. ≤−1.2089	149	0.825 [0.36; 1.88]	0.6466			
Puberty onset at inclusion (yes/no)						
Yes vs. no	291	0.433 [0.13; 1.47]	0.1805			
Bone age at treatment initiation (years)						
Median: >7 vs. ≤7	114	0.834 [0.30; 2.29]	0.7253			
GH dose at inclusion (mg/kg/day)						
Median: >0.0392 vs. ≤0.0392	273	1.354 [0.70; 2.61]	0.3643			
GH dose at last visit (mg/kg/day)						
Median >0.0406 vs. ≤0.0406	288	0.659 [0.35; 1.25]	0.2002			
IGF-I SDS at inclusion			0.3255			
>+2 SDS vs. <−2SDS	101	0.429 [0.07; 2.54]	0.3509			
−2 SDS to +2 SDS vs. <−2SDS	101	0.305 [0.06; 1.44]	0.1341			
Birth height SDS						
Median: >−2.4528 vs. ≤−2.4528	269	0.628 [0.32; 1.23]	0.1731			
Birth weight SDS						
Median: >−1.7643 vs. ≤−1.7643	278	0.617 [0.32; 1.18]	0.1452			
Registry participation while receiving treatment (years)						
Median: >4.9405 vs. ≤4.9405	266	1.872 [0.92; 3.80]	0.0829			
Cumulative dose during first 2 years of treatment (mg/kg)			0.7997			
25.5675 ± 10% vs. >28.12425	266	1.298 [0.58; 2.88]	0.5210			
<23.01075 vs. >28.12425	266	1.190 [0.53; 2.69]	0.6757			
Parameter	Univariate analysis			Multivariate analysis		
	n used	OR [95% CI]	P-value	n used	OR [95% CI]	P-value
Factors associated with experiencing an SAE						
Bone maturation at inclusion (years)						
Median: >1.5106 vs. ≤1.5106	114	0.585 [0.21; 1.64]	0.3077			
BMI at inclusion (kg/m^2^)						
Median: >14.8739 vs. ≤14.8739	276	0.758 [0.39; 1.46]	0.4075			

A univariate analysis using a logistic regression model of each of the parameters listed above separately was performed. All prognostic factors that demonstrated associations with the outcome <20% were included in the multivariate model. For multivariate analyses, a backward stepwise selection was used to remove nonsignificant variables (p≥0.05). The selection variables were stopped when no more variables could be removed from the model. At the end of this selection, the final model was obtained. AE, adverse event; BMI, body mass index; CI, confidence interval; GH, growth hormone; IGF-I, insulin-like growth factor-I; OR, odds ratio; SAE, serious adverse event; SDS, standard deviation score.

Two determinants were identified in the univariate analysis as being associated with an increased risk of experiencing an SAE: use of concomitant treatment (p<0.0001, OR = 4.30) and presence of chronic disease (p=0.0004, OR = 4.01). Use of concomitant treatment was also identified in the multivariate analysis as being associated with an increased risk of experiencing an SAE (p=0.0001, OR = 3.96).

Regarding the number of AEs, longer registry participation while receiving treatment (p=0.0018, OR = 1.70) was the only determinant identified as being significantly associated with an increased number of reported nonserious AEs in both the univariate and multivariate analyses (**Supplementary Table 5**).

Two determinants were associated with an increased number of reported SAEs: use of concomitant treatment (p<0.0001, OR = 3.84) and presence of chronic disease (p=0.0008, OR = 2.87). In the multivariate analysis, only use of concomitant treatment (p<0.0001, OR = 4.36) was significantly associated with an increased number of SAEs.

### 3.6 AEs of specific interest

There were six AEs of specific interest (relating to tumor, cardiovascular, and cerebrovascular pathologies) occurring in five patients (**Table 5**). Two events of tumor occurrence were reported in two patients: a malignant tumor (nephroblastoma) and a benign tumor (renal cysts). Malignant nephroblastoma with liver and lung metastases was reported as an SAE in a treatment-naïve patient who had been treated with GH for 3.5 years. Although the relation between the event and GH treatment was considered unlikely, GH treatment was stopped after tumor diagnosis. Following surgery and radiotherapy, further metastatic lesions of the liver were diagnosed. The patient died 1 year and 2 months after nephroblastoma diagnosis. No underlying etiology potentially associated with an increased risk for developing nephroblastoma was reported by the patient’s physician and causality of death was assessed as unlikely. A benign tumor (two cysts on the right kidney) was reported in a previously treated patient for whom the total duration of GH treatment was unknown. The event was not considered serious and the relationship to GH treatment was considered impossible to specify. No change in GH treatment was made and the event stabilized at the end of the study. In both patients with diagnosis of tumor, mean IGF-I SDS >+2 was reported three times between study inclusion and tumor diagnosis.

**Table 5:** Adverse events of specific interest (tumor, cardiovascular, and cerebrovascular pathologies [six events in five patients]).

	Treatment status	Presence of chronic disease	Age at event (years)	Age at treatment initiation (years)	Treatment duration at event (years)	GH dose at event (mg/kg/day)	Intensity	Serious (yes/no)	GH treatment change	Outcome	Relatedness to GH treatment
Tumors											
Malignant nephroblastoma with liver and lung metastases	Naïve	No	9.4	5.9	3.5	0.031	Severe	Yes	Stopped	Fatal	Unlikely
Two cysts on the right kidney (benign)	Previously treated	No	5.3	3.5	–	0.042	Mild	No	No change	Stabilized	Impossible to specify
Cardiovascular disorders											
Hypertrophic cardiomyopathy of the left ventricle	Previously treated	Yes	12.8	6.2	–	0.048	Severe	No	No change	Ongoing at study end	Unlikely
Tricuspid valve incompetence	Naïve	Yes	4.6	4.3	0.2	0.054	Moderate	Yes	No change	Stabilized	Unlikely
Cerebrovascular disorders											
Cerebral ventricular shunt for treatment of hydrocephalus*	Naïve	Yes	6.3	4.5	1.8	0.044	Moderate	Yes	No change	Resolved	Unlikely
Valve dysfunction with superinfection*	Naïve	Yes	7.2	4.5	2.7	0.056	–	Yes	No change	Resolved	Unlikely

*These events occurred in the same patient.

GH, growth hormone.

Two cardiac events were reported in two patients: one left ventricular hypertrophy in a previously treated patient with Silver Russell syndrome, and one tricuspid valve incompetence in a treatment-naïve patient with Goldenhar syndrome. Both events were considered unlikely to be related to GH treatment and neither resulted in GH treatment being altered or discontinued. At study end, the left ventricular hypertrophy remained ongoing, while the patient with tricuspid valve incompetence was reported as stabilized.

Lastly, one patient, diagnosed with Noonan syndrome, was treated with a ventriculo-cardiac shunt due to hydrocephalus at 6.3 years of age after treatment with GH for 1.8 years. The event was not considered related to treatment and GH dose was not adjusted following diagnosis. At 7.2 years of age, shunt dislocation with superinfection was reported in the same patient. Both events were reported as SAEs and neither event was considered related to GH treatment. For both events, GH treatment was not changed, and the outcomes were reported as resolved at study end.

## 4 Discussion

Using real-world data from a representative sample of children with short stature born SGA, we have explored the safety outcomes related to GH treatment in this population and analyzed the determinants significantly associated with these patients experiencing AEs and SAEs. We have shown that the occurrence of AEs was associated with the underlying conditions and concomitant treatments of these children born SGA. Overall, we report no concerning or unexpected safety findings. These data, in a large national cohort of children born SGA, are consistent with existing data which support a positive risk:benefit ratio for GH treatment (26).

The presence of chronic disease would be expected as a determinant as comorbidities are often associated with an increased AE profile. In this study, presence of chronic disease was a significant determinant for increased risk of experiencing an AE (p=0.0004, OR = 2.56) in the multivariate analysis. In addition, presence of chronic disease was also a significant determinant for both increased risk of onset (p=0.0004, OR = 4.010) and higher number (p=0.0008, OR = 2.873) of SAEs in the univariate analyses. Of note, concomitant treatment was a significant determinant for both the increased risk of onset and the higher number of SAEs, with a p-value of <0.0001 in both multivariate analyses. This may be explained by chronic disease being found to be a determinant for AEs and SAEs, and presence of chronic disease is likely to result in the use of concomitant treatment. GnRH analogues leuprorelin and triptorelin were identified as the most common concomitant medications, followed by salbutamol, levothyroxine, centrally acting sympathomimetics, and glucocorticoids.Determinants associated with risk of onset and number of AEs were reported. Longer registry participation while receiving treatment (>4.9 years) was identified as a determinant associated with an increased risk of experiencing an AE and an increased number of nonserious AEs. Longer treatment duration (≥7 years) was identified by Tidblad et al. (27) as a determinant of cardiovascular AEs. However, the authors caution that these patients may have been at underlying risk of cardiovascular disease for reasons other than GH treatment during childhood, such as the lack of continuous GH treatment during adulthood (27).

GH treatment has been shown to have a positive effect on blood pressure and lipid metabolism (28–30), but can also result in increased insulin resistance (27). Transient increases in insulin secretion and impaired insulin sensitivity have been reported previously in children born SGA receiving GH treatment (31–33). It is believed that this is a compensatory response to prevent hyperglycemia and may lead to an increased risk of developing type 2 diabetes. In the current long-term follow-up study, only one event of type 2 diabetes, one event of hyperinsulinemia, and one event of increased HbA1c were reported among the 291 patients.

Notably, of the six tumor, cardiovascular, and cerebrovascular pathologies defined as AEs of specific interest, none were considered possibly or probably related to GH treatment. AEs of specific interest occurred in both previously treated and treatment-naïve patients, and most patients had an underlying chronic disease. Furthermore, GH dose and GH treatment duration at time of event did not appear to be similar across patients experiencing a tumor, cardiovascular, or cerebrovascular event.

In the large SAGhE cohort study comprising 24,232 patients, long-term all-cause mortality after childhood growth hormone treatment was associated with the underlying diagnosis requiring rhGH treatment [9]. As part of the SAGhE study, the long-term risk of cancer incidence was analyzed for 10,406 patients treated with GH (19, 20). The patients had differing underlying conditions requiring GH treatment as well as variable daily GH doses. The data showed that there was no overall increase in cancer mortality or incidence risk with increasing cumulative GH dose or treatment duration (19, 20). However, for some patients with previous cancer, a link between cancer mortality and increasing daily GH doses was found (19). This may have been reflective of the conditions leading to GH therapy and their treatments (e.g. radiotherapy). As most cancers develop in adulthood, data from short-term studies provide limited information on cancer risk following childhood GH treatment and, therefore, large-scale, long-term follow-up studies on cancer risk remain of ongoing importance (19, 20).

Previous studies investigating the risk of cardiovascular events among patients born SGA treated with GH during childhood report that a causal relationship to GH treatment remains uncertain. Longer duration of GH treatment and higher cumulative GH dose may carry a risk, but it still remains low, if at all present (20, 27). Prolonged monitoring of patients treated with GH during childhood into later life is warranted. Additionally, the benefit of GH treatment should be carefully evaluated in children born SGA who are taking other medications.

Strengths of this observational, multicenter, noninterventional study include the representation of a large patient population and the broad, real-life data from clinical practice. However, the uncontrolled nature of data collection in observational studies may result in notable limitations. For instance, data completeness may be negatively impacted by incomplete or inaccurate reporting by physicians of patient data or confounding factors. The enrolment of selected clinics for this study may increase potential selection bias. Furthermore, the determination of AE or SAE relatedness to treatment by each patient’s treating physician may have been impacted by each physician’s subjective judgment. This study did not conduct analyses to assess determinants of rare adverse events (such as cardiovascular and cerebrovascular events) specifically, as the number of patients and patient-years of exposure were too limited to detect these events.

No new safety issues associated with GH therapy were identified during this study. Determining factors, which were significantly associated with risk of onset and number of AEs and SAEs, were longer registry participation while receiving treatment and the presence of chronic disease, with concomitant treatment as a significant determinant for both the onset and number of SAEs. Very few patients experienced events related to tumor, cardiovascular, and cerebrovascular pathologies, and there was no consistent trend in GH dose or GH treatment duration among these patients. These data contribute to the growing body of evidence that supports the good safety profile of GH therapy in pediatric patients with short stature born SGA, and may provide reassurance to clinicians treating this patient population. As such, data gathered from real-world studies continue to be useful for shaping clinical practice and optimizing treatment outcomes for children born SGA.

## Data Availability

The raw data supporting the conclusions of this article will be made available by the authors, without undue reservation.
