# Correction: Determinants of adverse event occurrence in children with short stature born small for gestational age treated with growth hormone

**DOI:** 10.3389/fendo.2025.1754016

**Published:** 2025-12-08

**Authors:** Regis Coutant, Nazim Benchikh, Agnès Linglart, Marc Nicolino, Jean-Pierre Salles

**Affiliations:** 1 Department of Paediatric Endocrinology, University Hospital, Angers, France; 2 Rare Endocrine Disorders, Clinical Medical and Regulatory, Novo Nordisk, Paris, France; 3 AP-HP, Paris Saclay University, INSERM, Physiologie et Physiopathologie Endocriniennes, Endocrinology and Diabetology for Children, Bicêtre Paris Saclay Hospital, Le Kremlin-Bicetre, France; 4 Division of Pediatric Endocrinology and Metabolism, Lyon University Pediatric Hospital, Lyon, France; 5 Endocrinology and Bone Diseases Unit, Children’s Hospital, Toulouse University Hospital, University of Toulouse Paul Sabatier, INSERM-CNRS Unit 1291, Infinity Center, Toulouse, France

**Keywords:** growth hormone, small for gestational age, adverse events, serious adverse events, real-world data

The reference for “23. World Medical Association (2004)” was erroneously written as “23. World Medical Association. Declaration of Helsinki: ethical principles for medical research involving human subjects. J Int Bioethique. (2004) 15:124–9. doi: 10.1001/jama.2013.281053”. It should be “23. World Medical Association. Declaration of Helsinki: ethical principles for medical research involving human subjects. J Int Bioethique. (2004) 15(1):124–9”.

The original version of this article has been updated.

