# Supplementary material for: Determinants of adverse event occurrence in children with short stature born small for gestational age treated with growth hormone
Source: Front Endocrinol (Lausanne). 2025 Nov 19;16:1656966. doi: 10.3389/fendo.2025.1656966 (PMC12672253; doi:10.3389/fendo.2025.1656966)
Supplement: Supplementary file 1 [file DataSheet1.pdf]

Supplementary Material

1. Supplementary Data

CONTENTS:

Supplementary Table 1. All reported nonserious AEs in the follow-up population, grouped by treatment status.

Supplementary Table 2. All reported SAEs in the follow-up population, grouped by treatment status.

Supplementary Table 3. AEs considered probably or possibly related to treatment in the follow-up population, grouped by treatment status.

Supplementary Table 4. SAEs considered probably or possibly related to treatment in the follow-up population, grouped by treatment.

Supplementary Table 5. Determinants associated with the number of AEs (univariate and multivariate logistic regression analyses).

Supplementary Table 1. All reported nonserious AEs in the follow-up population, grouped by treatment status.

	Previously treated (n=109)		Naïve (n=182)		Total (n=291)	
	Patients, n (%)	AEs, n (%)	Patients, n (%)	AEs, n (%)	Patients, n (%)	AEs, n (%)
**Patients with at least one nonserious AE**	**42 (38.53%)**		**81 (44.51%)**		**123 (42.27%)**	
**Number of nonserious AEs**		**57 (100.00%)**		**144 (100.00%)**		**201 (100.00%)**
**Blood and lymphatic system disorders**	**0 (0.00%)**	**0 (0.00%)**	**1 (0.55%)**	**1 (0.69%)**	**1 (0.34%)**	**1 (0.50%)**
Lymphadenitis	0 (0.00%)	0 (0.00%)	1 (0.55%)	1 (0.69%)	1 (0.34%)	1 (0.50%)
**Cardiac disorders**	**1 (0.92%)**	**1 (1.75%)**	**0 (0.00%)**	**0 (0.00%)**	**1 (0.34%)**	**1 (0.50%)**
Left ventricular hypertrophy	1 (0.92%)	1 (1.75%)	0 (0.00%)	0 (0.00%)	1 (0.34%)	1 (0.50%)
**Ear and labyrinth disorders**	**1 (0.92%)**	**1 (1.75%)**	**0 (0.00%)**	**0 (0.00%)**	**1 (0.34%)**	**1 (0.50%)**
Ear pain	1 (0.92%)	1 (1.75%)	0 (0.00%)	0 (0.00%)	1 (0.34%)	1 (0.50%)
**Endocrine disorders**	**1 (0.92%)**	**1 (1.75%)**	**0 (0.00%)**	**0 (0.00%)**	**1 (0.34%)**	**1 (0.50%)**
Hypothyroidism	1 (0.92%)	1 (1.75%)	0 (0.00%)	0 (0.00%)	1 (0.34%)	1 (0.50%)
**Gastrointestinal disorders**	**1 (0.92%)**	**1 (1.75%)**	**5 (2.75%)**	**5 (3.47%)**	**6 (2.06%)**	**6 (2.99%)**
Abdominal pain	1 (0.92%)	1 (1.75%)	2 (1.01%)	2 (1.39%)	3 (1.03%)	3 (1.49%)
Constipation	0 (0.00%)	0 (0.00%)	1 (0.55%)	1 (0.69%)	1 (0.34%)	1 (0.50%)
Dyspepsia	0 (0.00%)	0 (0.00%)	1 (0.55%)	1 (0.69%)	1 (0.34%)	1 (0.50%)
Teething	0 (0.00%)	0 (0.00%)	1 (0.55%)	1 (0.69%)	1 (0.34%)	1 (0.50%)
**General disorders and administration-site conditions**	**4 (3.67%)**	**4 (7.02%)**	**9 (4.95%)**	**10 (6.94%)**	**13 (4.47%)**	**14 (6.97%)**
Chest pain	0 (0.00%)	0 (0.00%)	1 (0.55%)	1 (0.69%)	1 (0.34%)	1 (0.50%)
Drug intolerance	1 (0.92%)	1 (1.75%)	0 (0.00%)	0 (0.00%)	1 (0.34%)	1 (0.50%)
Injection-site atrophy	0 (0.00%)	0 (0.00%)	2 (1.10%)	2 (1.39%)	2 (0.69%)	2 (1.00%)
Injection-site hematoma	1 (0.92%)	1 (1.75%)	2 (1.10%)	3 (2.08%)	3 (1.03%)	4 (1.99%)
Injection-site hemorrhage	1 (0.92%)	1 (1.75%)	1 (0.55%)	1 (0.69%)	2 (0.69%)	2 (1.00%)
Injection-site nodule	0 (0.00%)	0 (0.00%)	1 (0.55%)	1 (0.69%)	1 (0.34%)	1 (0.50%)
Injection-site pain	1 (0.92%)	1 (1.75%)	0 (0.00%)	0 (0.00%)	1 (0.34%)	1 (0.50%)
Injection-site pruritus	0 (0.00%)	0 (0.00%)	1 (0.55%)	1 (0.69%)	1 (0.34%)	1 (0.50%)
Malaise	0 (0.00%)	0 (0.00%)	1 (0.55%)	1 (0.69%)	1 (0.34%)	1 (0.50%)
**Infections and infestations**	**1 (0.92%)**	**1 (1.75%)**	**4 (2.20%)**	**5 (3.47%)**	**5 (1.72%)**	**6 (2.99%)**
Eczema infected	0 (0.00%)	0 (0.00%)	1 (0.55%)	1 (0.69%)	1 (0.34%)	1 (0.50%)
Fungal infection	0 (0.00%)	0 (0.00%)	1 (0.55%)	1 (0.69%)	1 (0.34%)	1 (0.50%)
Herpes zoster	1 (0.92%)	1 (1.75%)	0 (0.00%)	0 (0.00%)	1 (0.34%)	1 (0.50%)
Infectious mononucleosis	0 (0.00%)	0 (0.00%)	1 (0.55%)	1 (0.69%)	1 (0.34%)	1 (0.50%)
Nasopharyngitis	0 (0.00%)	0 (0.00%)	1 (0.55%)	1 (0.69%)	1 (0.34%)	1 (0.50%)
Tracheitis	0 (0.00%)	0 (0.00%)	1 (0.55%)	1 (0.69%)	1 (0.34%)	1 (0.50%)
**Injury, poisoning, and procedural complications**	**0 (0.00%)**	**0 (0.00%)**	**5 (2.75%)**	**5 (3.47%)**	**5 (1.72%)**	**5 (2.49%)**
Clavicle fracture	0 (0.00%)	0 (0.00%)	1 (0.55%)	1 (0.69%)	1 (0.34%)	1 (0.50%)

Incorrect dose administered	0 (0.00%)	0 (0.00%)	1 (0.55%)	1 (0.69%)	1 (0.34%)	1 (0.50%)
Joint injury	0 (0.00%)	0 (0.00%)	1 (0.55%)	1 (0.69%)	1 (0.34%)	1 (0.50%)
Radial head dislocation	0 (0.00%)	0 (0.00%)	1 (0.55%)	1 (0.69%)	1 (0.34%)	1 (0.50%)
Vaccination complication	0 (0.00%)	0 (0.00%)	1 (0.55%)	1 (0.69%)	1 (0.34%)	1 (0.50%)
**Investigations**	**17 (15.60%)**	**19 (33.33%)**	**37 (20.33%)**	**49 (34.03%)**	**54 (18.56%)**	**68 (33.83%)**
Blood creatine phosphokinase increased	0 (0.00%)	0 (0.00%)	1 (0.55%)	1 (0.69%)	1 (0.34%)	1 (0.50%)
Blood thyroid-stimulating hormone increased	1 (0.92%)	1 (1.75%)	1 (0.55%)	1 (0.69%)	2 (0.69%)	2 (1.00%)
Glycosylated hemoglobin increased	0 (0.00%)	0 (0.00%)	1 (0.55%)	1 (0.69%)	1 (0.34%)	1 (0.50%)
Insulin-like growth factor decreased	0 (0.00%)	0 (0.00%)	1 (0.55%)	1 (0.69%)	1 (0.34%)	1 (0.50%)
Insulin-like growth factor increased	17 (15.60%)	18 (31.58%)	33 (18.13%)	41 (28.47%)	50 (17.18%)	59 (29.35%)
Lymphocyte count increased	0 (0.00%)	0 (0.00%)	1 (0.55%)	1 (0.69%)	1 (0.34%)	1 (0.50%)
Neutrophil count decreased	0 (0.00%)	0 (0.00%)	1 (0.55%)	1 (0.69%)	1 (0.34%)	1 (0.50%)
Platelet count increased	0 (0.00%)	0 (0.00%)	1 (0.55%)	1 (0.69%)	1 (0.34%)	1 (0.50%)
Thyroxine decreased	0 (0.00%)	0 (0.00%)	1 (0.55%)	1 (0.69%)	1 (0.34%)	1 (0.50%)
**Metabolism and nutrition disorders**	**1 (0.92%)**	**1 (1.75%)**	**3 (1.65%)**	**3 (2.08%)**	**4 (1.37%)**	**4 (1.99%)**
Decreased appetite	0 (0.00%)	0 (0.00%)	1 (0.55%)	1 (0.69%)	1 (0.34%)	1 (0.50%)
Fluid retention	0 (0.00%)	0 (0.00%)	1 (0.55%)	1 (0.69%)	1 (0.34%)	1 (0.50%)
Hyperinsulinemia	0 (0.00%)	0 (0.00%)	1 (0.55%)	1 (0.69%)	1 (0.34%)	1 (0.50%)
Mitochondrial cytopathy	1 (0.92%)	1 (1.75%)	0 (0.00%)	0 (0.00%)	1 (0.34%)	1 (0.50%)
**Musculoskeletal and connective tissue disorders**	**10 (9.17%)**	**12 (21.05%)**	**16 (8.79%)**	**19 (13.19%)**	**26 (8.93%)**	**31 (15.42%)**
Arthralgia	8 (7.34%)	8 (14.04%)	5 (2.75%)	6 (4.17%)	13 (4.47%)	14 (6.97%)
Back pain	0 (0.00%)	0 (0.00%)	2 (1.10%)	2 (1.39%)	2 (0.69%)	2 (1.00%)
Exostosis	0 (0.00%)	0 (0.00%)	1 (0.55%)	1 (0.69%)	1 (0.34%)	1 (0.50%)
Foot deformity	0 (0.00%)	0 (0.00%)	1 (0.55%)	1 (0.69%)	1 (0.34%)	1 (0.50%)
Kyphosis	0 (0.00%)	0 (0.00%)	1 (0.55%)	1 (0.69%)	1 (0.34%)	1 (0.50%)
Ligament laxity	0 (0.00%)	0 (0.00%)	1 (0.55%)	1 (0.69%)	1 (0.34%)	1 (0.50%)
Muscle disorder	0 (0.00%)	0 (0.00%)	1 (0.55%)	1 (0.69%)	1 (0.34%)	1 (0.50%)
Myalgia	2 (1.83%)	2 (3.51%)	0 (0.00%)	0 (0.00%)	2 (0.69%)	2 (1.00%)
Osteochondritis	1 (0.92%)	1 (1.75%)	0 (0.00%)	0 (0.00%)	1 (0.34%)	1 (0.50%)
Pain in extremity	0 (0.00%)	0 (0.00%)	5 (2.75%)	5 (3.47%)	5 (1.72%)	5 (2.49%)
Scoliosis	0 (0.00%)	0 (0.00%)	1 (0.55%)	1 (0.69%)	1 (0.34%)	1 (0.50%)
Spinal pain	1 (0.92%)	1 (1.75%)	0 (0.00%)	0 (0.00%)	1 (0.34%)	1 (0.50%)
**Musculoskeletal and connective tissue disorders; musculoskeletal and connective tissue disorders**	**0 (0.00%)**	**0 (0.00%)**	**1 (0.55%)**	**1 (0.69%)**	**1 (0.34%)**	**1 (0.50%)**
Arthralgia	0 (0.00%)	0 (0.00%)	1 (0.55%)	1 (0.69%)	1 (0.34%)	1 (0.50%)
**Nervous system disorders**	**4 (3.67%)**	**4 (7.02%)**	**27 (14.74%)**	**27 (18.75%)**	**31 (10.65%)**	**31 (15.42%)**

Dizziness	0 (0.00%)	0 (0.00%)	1 (0.55%)	1 (0.69%)	1 (0.34%)	1 (0.50%)
Headache	3 (2.75%)	3 (5.26%)	24 (13.19%)	24 (16.67%)	27 (9.28%)	27 (13.43%)
Migraine	0 (0.00%)	0 (0.00%)	1 (0.55%)	1 (0.69%)	1 (0.34%)	1 (0.50%)
Presyncope	1 (0.92%)	1 (1.75%)	0 (0.00%)	0 (0.00%)	1 (0.34%)	1 (0.50%)
Tension headache	0 (0.00%)	0 (0.00%)	1 (0.55%)	1 (0.69%)	1 (0.34%)	1 (0.50%)
**Psychiatric disorders**	**0 (0.00%)**	**0 (0.00%)**	**6 (3.30%)**	**7 (4.86%)**	**6 (2.06%)**	**7 (3.48%)**
Abnormal behavior	0 (0.00%)	0 (0.00%)	2 (1.10%)	2 (1.39%)	2 (0.69%)	2 (1.00%)
Aggression	0 (0.00%)	0 (0.00%)	2 (1.10%)	2 (1.39%)	2 (0.69%)	2 (1.00%)
Agitation	0 (0.00%)	0 (0.00%)	2 (1.10%)	2 (1.39%)	2 (0.69%)	2 (1.00%)
Attention-deficit/hyperactivity disorder	0 (0.00%)	0 (0.00%)	1 (0.55%)	1 (0.69%)	1 (0.34%)	1 (0.50%)
**Renal and urinary disorders**	**2 (1.83%)**	**2 (3.51%)**	**0 (0.00%)**	**0 (0.00%)**	**2 (0.69%)**	**2 (1.00%)**
Hypertonic bladder	1 (0.92%)	1 (1.75%)	0 (0.00%)	0 (0.00%)	1 (0.34%)	1 (0.50%)
Renal cyst	1 (0.92%)	1 (1.75%)	0 (0.00%)	0 (0.00%)	1 (0.34%)	1 (0.50%)
**Reproductive system and breast disorders**	**1 (0.92%)**	**1 (1.75%)**	**0 (0.00%)**	**0 (0.00%)**	**1 (0.34%)**	**1 (0.50%)**
Gynecomastia	1 (0.92%)	1 (1.75%)	0 (0.00%)	0 (0.00%)	1 (0.34%)	1 (0.50%)
**Respiratory, thoracic, and mediastinal disorders**	**3 (2.75%)**	**3 (5.26%)**	**5 (2.75%)**	**6 (4.17%)**	**8 (2.75%)**	**9 (4.48%)**
Asthma	1 (0.92%)	1 (1.75%)	4 (2.20%)	5 (3.47%)	5 (1.72%)	6 (2.99%)
Choking	1 (0.92%)	1 (1.75%)	0 (0.00%)	0 (0.00%)	1 (0.34%)	1 (0.50%)
Epistaxis	1 (0.92%)	1 (1.75%)	1 (0.55%)	1 (0.69%)	2 (0.69%)	2 (1.00%)
**Skin and subcutaneous tissue disorders**	**0 (0.00%)**	**0 (0.00%)**	**3 (1.65%)**	**3 (2.08%)**	**3 (1.03%)**	**3 (1.49%)**
Erythema	0 (0.00%)	0 (0.00%)	1 (0.55%)	1 (0.69%)	1 (0.34%)	1 (0.50%)
Scar pain	0 (0.00%)	0 (0.00%)	1 (0.55%)	1 (0.69%)	1 (0.34%)	1 (0.50%)
Vitiligo	0 (0.00%)	0 (0.00%)	1 (0.55%)	1 (0.69%)	1 (0.34%)	1 (0.50%)
**Surgical and medical procedures**	**4 (3.67%)**	**6 (10.53%)**	**2 (1.10%)**	**2 (1.39%)**	**6 (2.06%)**	**8 (3.98%)**
Adenotonsillectomy	0 (0.00%)	0 (0.00%)	1 (0.55%)	1 (0.69%)	1 (0.34%)	1 (0.50%)
Appendicectomy	1 (0.92%)	1 (1.75%)	0 (0.00%)	0 (0.00%)	1 (0.34%)	1 (0.50%)
Fasciotomy	1 (0.92%)	1 (1.75%)	0 (0.00%)	0 (0.00%)	1 (0.34%)	1 (0.50%)
Limb operation	0 (0.00%)	0 (0.00%)	1 (0.55%)	1 (0.69%)	1 (0.34%)	1 (0.50%)
Orchidopexy	1 (0.92%)	2 (3.51%)	0 (0.00%)	0 (0.00%)	1 (0.34%)	2 (1.00%)
Skin lesion removal	1 (0.92%)	1 (1.75%)	0 (0.00%)	0 (0.00%)	1 (0.34%)	1 (0.50%)
Tenotomy	1 (0.92%)	1 (1.75%)	0 (0.00%)	0 (0.00%)	1 (0.34%)	1 (0.50%)
**Vascular disorders**	**0 (0.00%)**	**0 (0.00%)**	**1 (0.55%)**	**1 (0.69%)**	**1 (0.34%)**	**1 (0.50%)**
Raynaud's phenomenon	0 (0.00%)	0 (0.00%)	1 (0.55%)	1 (0.69%)	1 (0.34%)	1 (0.50%)

AE, adverse event.

Supplementary Table 2. All reported SAEs in the follow-up population, grouped by treatment status.

	Previously treated (n=109)		Naïve (n=182)		Total (n=291)	
	Patients, n (%)	AEs, n (%)	Patients, n (%)	AEs, n (%)	Patients, n (%)	AEs, n (%)
**Patients with at least one serious event**	**17 (15.60%)**		**29 (15.93%)**		**46 (15.81%)**	
**Number of SAEs**		**27 (100.00%)**		**59 (100.00%)**		**86 (100.00%)**
**Cardiac disorders**	**0 (0.00%)**	**0 (0.00%)**	**1 (0.55%)**	**1 (1.69%)**	**1 (0.34%)**	**1 (1.16%)**
Tricuspid valve incompetence	0 (0.00%)	0 (0.00%)	1 (0.55%)	1 (1.69%)	1 (0.34%)	1 (1.16%)
**Congenital, familial, and genetic disorders**	2 (1.83%)	2 (7.41%)	2 (1.10%)	2 (3.39%)	4 (1.37%)	4 (4.65%)
Cryptorchism	1 (0.92%)	1 (3.70%)	1 (0.55%)	1 (1.69%)	2 (0.69%)	2 (2.33%)
Hypospadias	1 (0.92%)	1 (3.70%)	0 (0.00%)	0 (0.00%)	1 (0.34%)	1 (1.16%)
Pectus excavatum	0 (0.00%)	0 (0.00%)	1 (0.55%)	1 (1.69%)	1 (0.34%)	1 (1.16%)
**Gastrointestinal disorders**	**1 (0.92%)**	**1 (3.70%)**	**5 (2.75%)**	**5 (8.47%)**	**6 (2.06%)**	**6 (6.98%)**
Abdominal pain	0 (0.00%)	0 (0.00%)	1 (0.55%)	1 (1.69%)	1 (0.34%)	1 (1.16%)
Gastro-esophageal reflux disease	1 (0.92%)	1 (3.70%)	1 (0.55%)	1 (1.69%)	2 (0.69%)	2 (2.33%)
Nausea	0 (0.00%)	0 (0.00%)	1 (0.55%)	1 (1.69%)	1 (0.34%)	1 (1.16%)
Rectal hemorrhage	0 (0.00%)	0 (0.00%)	1 (0.55%)	1 (1.69%)	1 (0.34%)	1 (1.16%)
Vomiting	0 (0.00%)	0 (0.00%)	1 (0.55%)	1 (1.69%)	1 (0.34%)	1 (1.16%)
**General disorders and administration-site conditions**	**1 (0.92%)**	**1 (3.70%)**	**2 (1.10%)**	**2 (3.39%)**	**3 (1.03%)**	**3 (3.49%)**
Asthenia	1 (0.92%)	1 (3.70%)	0 (0.00%)	0 (0.00%)	1 (0.34%)	1 (1.16%)
Pyrexia	0 (0.00%)	0 (0.00%)	1 (0.55%)	1 (1.69%)	1 (0.34%)	1 (1.16%)
Swelling	0 (0.00%)	0 (0.00%)	1 (0.55%)	1 (1.69%)	1 (0.34%)	1 (1.16%)
**Infections and infestations**	**4 (3.67%)**	**5 (18.52%)**	**4 (2.20%)**	**9 (15.25%)**	**8 (2.75%)**	**14 (16.28%)**
Device related infection	0 (0.00%)	0 (0.00%)	1 (0.55%)	1 (1.69%)	1 (0.34%)	1 (1.16%)
Enterococcal sepsis	0 (0.00%)	0 (0.00%)	1 (0.55%)	1 (1.69%)	1 (0.34%)	1 (1.16%)
Gastroenteritis	2 (1.83%)	2 (7.41%)	2 (1.10%)	2 (3.39%)	4 (1.37%)	4 (4.65%)
Oral herpes	1 (0.92%)	1 (3.70%)	0 (0.00%)	0 (0.00%)	1 (0.34%)	1 (1.16%)
Pneumonia pneumococcal	1 (0.92%)	1 (3.70%)	0 (0.00%)	0 (0.00%)	1 (0.34%)	1 (1.16%)
Pyelonephritis	0 (0.00%)	0 (0.00%)	1 (0.55%)	3 (5.08%)	1 (0.34%)	3 (3.49%)
Pyelonephritis acute	0 (0.00%)	0 (0.00%)	1 (0.55%)	1 (1.69%)	1 (0.34%)	1 (1.16%)
Staphylococcal sepsis	0 (0.00%)	0 (0.00%)	1 (0.55%)	1 (1.69%)	1 (0.34%)	1 (1.16%)
Superinfection bacterial	1 (0.92%)	1 (3.70%)	0 (0.00%)	0 (0.00%)	1 (0.34%)	1 (1.16%)
**Injury, poisoning, and procedural complications**	**3 (2.75%)**	**4 (14.81%)**	**3 (1.65%)**	**3 (5.08%)**	**6 (2.06%)**	**7 (8.14%)**
Extradural hematoma	1 (0.92%)	1 (3.70%)	0 (0.00%)	0 (0.00%)	1 (0.34%)	1 (1.16%)
Gastrointestinal anastomotic leak	0 (0.00%)	0 (0.00%)	1 (0.55%)	1 (1.69%)	1 (0.34%)	1 (1.16%)
Head injury	0 (0.00%)	0 (0.00%)	1 (0.55%)	1 (1.69%)	1 (0.34%)	1 (1.16%)
Joint dislocation	1 (0.92%)	1 (3.70%)	0 (0.00%)	0 (0.00%)	1 (0.34%)	1 (1.16%)

Post procedural hematoma	0 (0.00%)	0 (0.00%)	1 (0.55%)	1 (1.69%)	1 (0.34%)	1 (1.16%)
Post procedural hemorrhage	1 (0.92%)	1 (3.70%)	0 (0.00%)	0 (0.00%)	1 (0.34%)	1 (1.16%)
Radial head dislocation	1 (0.92%)	1 (3.70%)	0 (0.00%)	0 (0.00%)	1 (0.34%)	1 (1.16%)
**Investigations**	**1 (0.92%)**	**1 (3.70%)**	**0 (0.00%)**	**0 (0.00%)**	**1 (0.34%)**	**1 (1.16%)**
Weight increased	1 (0.92%)	1 (3.70%)	0 (0.00%)	0 (0.00%)	1 (0.34%)	1 (1.16%)
**Metabolism and nutrition disorders**	**1 (0.92%)**	**1 (3.70%)**	**2 (1.10%)**	**2 (3.39%)**	**3 (1.03%)**	**3 (3.49%)**
Decreased appetite	0 (0.00%)	0 (0.00%)	1 (0.55%)	1 (1.69%)	1 (0.34%)	1 (1.16%)
Type 2 diabetes mellitus	1 (0.92%)	1 (3.70%)	0 (0.00%)	0 (0.00%)	1 (0.34%)	1 (1.16%)
Weight gain poor	0 (0.00%)	0 (0.00%)	1 (0.55%)	1 (1.69%)	1 (0.34%)	1 (1.16%)
**Musculoskeletal and connective tissue disorders**	**2 (1.83%)**	**2 (7.41%)**	**2 (1.10%)**	**3 (5.08%)**	**4 (1.37%)**	**5 (5.81%)**
Bone pain	1 (0.92%)	1 (3.70%)	0 (0.00%)	0 (0.00%)	1 (0.34%)	1 (1.16%)
Epiphysiolysis	0 (0.00%)	0 (0.00%)	1 (0.55%)	2 (3.39%)	1 (0.34%)	2 (2.33%)
Fistula	0 (0.00%)	0 (0.00%)	1 (0.55%)	1 (1.69%)	1 (0.34%)	1 (1.16%)
Osteoarthritis	1 (0.92%)	1 (3.70%)	0 (0.00%)	0 (0.00%)	1 (0.34%)	1 (1.16%)
**Neoplasms benign, malignant, and unspecified (including cysts and polyps)**	**0 (0.00%)**	**0 (0.00%)**	**1 (0.55%)**	**1 (1.69%)**	**1 (0.34%)**	**1 (1.16%)**
Nephroblastoma	0 (0.00%)	0 (0.00%)	1 (0.55%)	1 (1.69%)	1 (0.34%)	1 (1.16%)
**Psychiatric disorders**	**0 (0.00%)**	**0 (0.00%)**	**1 (0.55%)**	**1 (1.69%)**	**1 (0.34%)**	**1 (1.16%)**
Anxiety	0 (0.00%)	0 (0.00%)	1 (0.55%)	1 (1.69%)	1 (0.34%)	1 (1.16%)
**Renal and urinary disorders**	1 (0.92%)	1 (3.70%)	0 (0.00%)	0 (0.00%)	1 (0.34%)	1 (1.16%)
Hematuria	1 (0.92%)	1 (3.70%)	0 (0.00%)	0 (0.00%)	1 (0.34%)	1 (1.16%)
**Reproductive system and breast disorders**	**1 (0.92%)**	**1 (3.70%)**	**0 (0.00%)**	**0 (0.00%)**	**1 (0.34%)**	**1 (1.16%)**
Testicular infarction	1 (0.92%)	1 (3.70%)	0 (0.00%)	0 (0.00%)	1 (0.34%)	1 (1.16%)
**Respiratory, thoracic, and mediastinal disorders**	**1 (0.92%)**	**1 (3.70%)**	**7 (3.85%)**	**10 (16.95%)**	**8 (2.75%)**	**11 (12.79%)**
Acute respiratory distress syndrome	1 (0.92%)	1 (3.70%)	1 (0.55%)	2 (3.39%)	2 (0.69%)	3 (3.49%)
Asthma	0 (0.00%)	0 (0.00%)	3 (1.65%)	3 (5.08%)	3 (1.03%)	3 (3.49%)
Dyspnea	0 (0.00%)	0 (0.00%)	1 (0.55%)	2 (3.39%)	1 (0.34%)	2 (2.33%)
Lung disorder	0 (0.00%)	0 (0.00%)	1 (0.55%)	2 (3.39%)	1 (0.34%)	2 (2.33%)
Sleep apnea syndrome	0 (0.00%)	0 (0.00%)	1 (0.55%)	1 (1.69%)	1 (0.34%)	1 (1.16%)
**Skin and subcutaneous tissue disorders**	**1 (0.92%)**	**1 (3.70%)**	**0 (0.00%)**	**0 (0.00%)**	**1 (0.34%)**	**1 (1.16%)**
Acanthosis nigricans	1 (0.92%)	1 (3.70%)	0 (0.00%)	0 (0.00%)	1 (0.34%)	1 (1.16%)
**Surgical and medical procedures**	6 (5.50%)	6 (22.22%)	11 (6.04%)	20 (33.90%)	17 (5.84%)	26 (30.23%)
Adenoidectomy	0 (0.00%)	0 (0.00%)	1 (0.55%)	1 (1.69%)	1 (0.34%)	1 (1.16%)
Adenotonsillectomy	0 (0.00%)	0 (0.00%)	1 (0.55%)	1 (1.69%)	1 (0.34%)	1 (1.16%)
Amygdalotomy	0 (0.00%)	0 (0.00%)	1 (0.55%)	1 (1.69%)	1 (0.34%)	1 (1.16%)
Appendicectomy	0 (0.00%)	0 (0.00%)	4 (2.20%)	4 (6.78%)	4 (1.37%)	4 (4.65%)
Bunion operation	1 (0.92%)	1 (3.70%)	0 (0.00%)	0 (0.00%)	1 (0.34%)	1 (1.16%)

Cast application	0 (0.00%)	0 (0.00%)	1 (0.55%)	1 (1.69%)	1 (0.34%)	1 (1.16%)
Ear tube insertion	0 (0.00%)	0 (0.00%)	1 (0.55%)	1 (1.69%)	1 (0.34%)	1 (1.16%)
Enteral nutrition	0 (0.00%)	0 (0.00%)	1 (0.55%)	1 (1.69%)	1 (0.34%)	1 (1.16%)
Eyelid operation	1 (0.92%)	1 (3.70%)	0 (0.00%)	0 (0.00%)	1 (0.34%)	1 (1.16%)
Facial operation	1 (0.92%)	1 (3.70%)	0 (0.00%)	0 (0.00%)	1 (0.34%)	1 (1.16%)
Gastrostomy	0 (0.00%)	0 (0.00%)	1 (0.55%)	1 (1.69%)	1 (0.34%)	1 (1.16%)
Hip arthroplasty	0 (0.00%)	0 (0.00%)	1 (0.55%)	1 (1.69%)	1 (0.34%)	1 (1.16%)
Hospitalization	1 (0.92%)	1 (3.70%)	0 (0.00%)	0 (0.00%)	1 (0.34%)	1 (1.16%)
Limb operation	1 (0.92%)	1 (3.70%)	0 (0.00%)	0 (0.00%)	1 (0.34%)	1 (1.16%)
Medical device change	0 (0.00%)	0 (0.00%)	1 (0.55%)	1 (1.69%)	1 (0.34%)	1 (1.16%)
Orchidopexy	0 (0.00%)	0 (0.00%)	1 (0.55%)	1 (1.69%)	1 (0.34%)	1 (1.16%)
Otoplasty	0 (0.00%)	0 (0.00%)	1 (0.55%)	1 (1.69%)	1 (0.34%)	1 (1.16%)
Pyeloplasty	1 (0.92%)	1 (3.70%)	0 (0.00%)	0 (0.00%)	1 (0.34%)	1 (1.16%)
Talipes correction	0 (0.00%)	0 (0.00%)	1 (0.55%)	1 (1.69%)	1 (0.34%)	1 (1.16%)
Tooth extraction	0 (0.00%)	0 (0.00%)	1 (0.55%)	1 (1.69%)	1 (0.34%)	1 (1.16%)
Tracheal plastic repair	0 (0.00%)	0 (0.00%)	1 (0.55%)	1 (1.69%)	1 (0.34%)	1 (1.16%)
Ventriculo-cardiac shunt	0 (0.00%)	0 (0.00%)	1 (0.55%)	1 (1.69%)	1 (0.34%)	1 (1.16%)
Vesicoureteral reflux surgery	0 (0.00%)	0 (0.00%)	1 (0.55%)	1 (1.69%)	1 (0.34%)	1 (1.16%)

AE, adverse event; SAE, serious adverse event.

frontiers

Supplementary Table 3. AEs considered probably or possibly related to treatment in the follow-up population, grouped by treatment status.

Parameter	Previously treated (n=109)		Treatment-naïve (n=182)		Total (n=291)	
	Patients (%)	AEs (%)	Patients (%)	AEs (%)	Patients (%)	AEs (%)
**Patients with ≥ 1 AE/number of AEs**	**32 (29.36)**	**34 (30.36)**	**56 (30.77)**	**78 (69.64)**	**88 (30.24)**	**112 (100.00)**
**Endocrine disorders**	1 (3.13)	1 (2.94)	0	0	1 (1.14)	1 (0.89)
Hypothyroidism	1 (3.13)	1 (2.94)	0	0	1 (1.14)	1 (0.89)
**General disorders and administration-site conditions**	4 (12.50)	4 (11.76)	7 (12.50)	7 (8.97)	11 (12.50)	11 (9.82)
Chest pain	0	0	1 (1.79)	1 (1.28)	1 (1.14)	1 (0.89)
Drug intolerance	1 (3.13)	1 (2.94)	0	0	1 (1.14)	1 (0.89)
Injection-site atrophy	0	0	2 (3.57)	2 (2.56)	2 (2.27)	2 (1.79)
Injection-site hematoma	1 (3.13)	1 (2.94)	1 (1.79)	1 (1.28)	2 (2.27)	2 (1.79)
Injection-site hemorrhage	1 (3.13)	1 (2.94)	1 (1.79)	1 (1.28)	2 (2.27)	2 (1.79)
Injection-site nodule	0	0	1 (1.79)	1 (1.28)	1 (1.14)	1 (0.89)
Injection-site pain	1 (3.13)	1 (2.94)	0	0	1 (1.14)	1 (0.89)
Injection-site pruritus	0	0	1 (1.79)	1 (1.28)	1 (1.14)	1 (0.89)
**Infections and infestations**	0	0	1 (1.79)	1 (1.28)	1 (1.14)	1 (0.89)
Nasopharyngitis	0	0	1 (1.79)	1 (1.28)	1 (1.14)	1 (0.89)
**Investigations**	17 (53.13)	18 (52.94)	32 (57.14)	39 (50.00)	49 (55.68)	57 (50.89)
Blood thyroid-stimulating hormone increased	0	0	1 (1.79)	1 (1.28)	1 (1.14)	1 (0.89)
Glycosylated hemoglobin increased	0	0	1 (1.79)	1 (1.28)	1 (1.14)	1 (0.89)
Insulin-like growth factor decreased	0	0	1 (1.79)	1 (1.28)	1 (1.14)	1 (0.89)
Insulin-like growth factor increased	17 (53.13)	18 (52.94)	30 (53.57)	36 (46.15)	47 (53.41)	54 (48.21)
**Metabolism and nutrition disorders**	1 (3.13)	1 (2.94)	1 (1.79)	1 (1.28)	2 (2.27)	2 (1.79)
Hyperinsulinemia	0	0	1 (1.79)	1 (1.28)	1 (1.14)	1 (0.89)
Type 2 diabetes mellitus	1 (3.13)	1 (2.94)	0	0	1 (1.14)	1 (0.89)
**Musculoskeletal and connective tissue disorders**	6 (18.75)	7 (20.59)	9 (16.07)	11 (14.10)	15 (17.05)	18 (16.07)
Arthralgia	4 (12.50)	4 (11.76)	3 (5.36)	3 (3.85)	7 (7.95)	7 (6.25)
Back pain	0	0	1 (1.79)	1 (1.28)	1 (1.14)	1 (0.89)
Epiphysiolysis	0	0	1 (1.79)	2 (2.56)	1 (1.14)	2 (1.79)
Kyphosis	0	0	1 (1.79)	1 (1.28)	1 (1.14)	1 (0.89)
Muscle disorder	0	0	1 (1.79)	1 (1.28)	1 (1.14)	1 (0.89)
Myalgia	1 (3.13)	1 (2.94)	0	0	1 (1.14)	1 (0.89)
Osteochondritis	1 (3.13)	1 (2.94)	0	0	1 (1.14)	1 (0.89)
Pain in extremity	0	0	3 (5.36)	3 (3.85)	3 (3.41)	3 (2.68)
Spinal pain	1 (3.13)	1 (2.94)	0	0	1 (1.14)	1 (0.89)

Parameter	Previously treated (n=109)		Treatment-naïve (n=182)		Total (n=291)	
	Patients (%)	AEs (%)	Patients (%)	AEs (%)	Patients (%)	AEs (%)
**Musculoskeletal and connective tissue disorders; musculoskeletal and connective tissue disorders**	0	0	1 (1.79)	1 (1.28)	1 (1.14)	1 (0.89)
Arthralgia	0	0	1 (1.79)	1 (1.28)	1 (1.14)	1 (0.89)
**Nervous system disorders**	0	0	12 (21.43)	12 (15.38)	12 (13.64)	12 (10.71)
Dizziness	0	0	1 (1.79)	1 (1.28)	1 (1.14)	1 (0.89)
Headache	0	0	10 (17.86)	10 (12.82)	10 (11.36)	10 (8.93)
Migraine	0	0	1 (1.79)	1 (1.28)	1 (1.14)	1 (0.89)
**Psychiatric disorders**	0	0	1 (1.79)	1 (1.28)	1 (1.14)	1 (0.89)
Attention-deficit/hyperactivity disorder	0	0	1 (1.79)	1 (1.28)	1 (1.14)	1 (0.89)
**Reproductive system and breast disorders**	2 (6.25)	2 (5.88)	0	0	2 (2.27)	2 (1.79)
Gynecomastia	1 (3.13)	1 (2.94)	0	0	1 (1.14)	1 (0.89)
Testicular infarction	1 (3.13)	1 (2.94)	0	0	1 (1.14)	1 (0.89)
**Respiratory, thoracic, and mediastinal disorders**	1 (3.13)	1 (2.94)	1 (1.79)	3 (3.85)	2 (2.27)	4 (3.57)
Asthma	1 (3.13)	1 (2.94)	1 (1.79)	3 (3.85)	2 (2.27)	4 (3.57)
**Skin and subcutaneous tissue disorders**	0	0	1 (1.79)	1 (1.28)	1 (1.14)	1 (0.89)
Erythema	0	0	1 (1.79)	1 (1.28)	1 (1.14)	1 (0.89)
**Surgical and medical procedures**	0	0	1 (1.79)	1 (1.28)	1 (1.14)	1 (0.89)
Hip arthroplasty	0	0	1 (1.79)	1 (1.28)	1 (1.14)	1 (0.89)

AE, adverse event.

Supplementary Table 4. SAEs considered probably or possibly related to treatment in the follow-up population, grouped by treatment status.

Parameter	Previously treated (n=109)		Naive (n=182)		Total (n=291)	
	Patients (%)	SAEs (%)	Patients (%)	SAEs (%)	Patients (%)	SAEs (%)
**SAEs possibly or probably related to treatment**	**2 (1.83)**	**2 (33.33)**	**2 (1.10)**	**4 (66.67)**	**4 (1.37)**	**6 (100.00)**
Type 2 diabetes mellitus	1 (50.00)	1 (50.00)	0	0	1 (25.00)	1 (16.67)
Epiphysiolysis	0	0	1 (50.00)	2 (50.00)	1 (25.00)	2 (33.33)
Testicular infarction	1 (50.00)	1 (50.00)	0	0	1 (25.00)	1 (16.67)
Asthma	0	0	1 (50.00)	1 (25.00)	1 (25.00)	1 (16.67)
Hip arthroplasty	0	0	1 (50.00)	1 (25.00)	1 (25.00)	1 (16.67)

One patient may have experienced more than one SAE.
SAE, serious adverse event.

Supplementary Table 5. Determinants associated with the number of AEs (univariate and multivariate logistic regression analyses).

Parameter	Univariate analysis			Multivariate analysis		
	n used	OR [95% CI]	P-value	n used	OR [95% CI]	P-value
**Factors associated with the number of nonserious AEs**						
**Registry participation while receiving treatment (years)**						
Median: >4.9405 vs. ≤4.9405	266	1.703 [1.22; 2.38]	0.0018	266	1.703 [1.22; 2.28]	0.0018
**Chronic diseases (yes/no)**						
Yes vs. no	291	1.324 [0.97; 1.80]	0.0732			
**Puberty onset at inclusion (yes/no)**						
Yes vs. no	291	0.525 [0.30; 0.92]	0.0246			
**Bone age at treatment initiation (years)**						
Median: >7 vs. ≤7	114	0.503 [0.30; 0.86]	0.0114			
**Height SDS at inclusion**						
>−3 vs. ≤−3	277	0.844 [0.61; 1.16]	0.2975			
**Height SDS at last visit**						
Median: >−2.4627 vs. ≤−2.4627	291	1.186 [0.88; 1.60]	0.2664			
**Height velocity at treatment initiation (SDS/year)**						
Median: >−1.2089 vs. ≤−1.2089	149	0.806 [0.53; 1.22]	0.3097			
**GH dose at inclusion (mg/kg/day)**						
Median: >0.0392 vs. ≤0.0392	273	0.997 [0.73; 1.36]	0.9847			
**GH dose at last visit (mg/kg/day)**						
Median: >0.0406 vs. ≤0.0406	288	0.753 [0.56; 1.02]	0.0675			
**IGF-I SDS at inclusion**			0.8824			
>+2 SDS vs. <−2SDS	101	1.076 [0.43; 2.71]	0.8762			
−2 SDS to +2 SDS vs. <−2SDS	101	0.920 [0.40; 2.11]	0.8445			
**Birth height SDS**						
Median: >−2.4528 vs. ≤−2.4528	269	1.045 [0.76; 1.43]	0.7842			
**Birth weight SDS**						
Median: >−1.7643 vs. ≤−1.7643	278	1.089 [0.80; 1.48]	0.5856			
**Chronological age at treatment initiation (years)**						
Median: >5.9863 vs. ≤5.9863	288	0.921 [0.68; 1.24]	0.5927			
**Cumulative dose during first 2 years of treatment (mg/kg)**			0.8518			
25.5675 ± 10% vs. >28.12425	266	1.103 [0.75; 1.61]	0.6139			
<23.01075 vs. >28.12425	266	0.988 [0.67; 1.46]	0.9499			
**Bone maturation at inclusion (years)**						
Median: >1.5106 vs. ≤1.5106	114	1.172 [0.71; 1.92]	0.5300			

Parameter	Univariate analysis			Multivariate analysis		
	n used	OR [95% CI]	P-value	n used	OR [95% CI]	P-value
**Concomitant treatment (yes/no)**						
Yes vs. no	291	1.076 [0.79; 1.46]	0.6363			
**BMI at inclusion (kg/m^2)**						
Median: >14.8739 vs. ≤ 14.8739	276	0.894 [0.66; 1.22]	0.4781			
**Factors associated with the number of SAEs**						
**Concomitant treatment (yes/no)**						
Yes vs. no	291	3.837 [2.14; 6.87]	<0.0001	269	4.361 [2.33; 8.15]	<0.0001
**Height SDS at inclusion**						
>−3 vs. ≤−3	277	0.531 [0.33; 0.86]	0.0094			
**Birth height SDS**						
Median: >−2.4528 vs. ≤−2.4528	269	0.597 [0.36; 0.99]	0.0440	269	0.525 [0.31; 0.90]	0.0196
**Chronic diseases (yes/no)**						
Yes vs. no	291	2.873 [1.55; 5.31]	0.0008			
**Chronological age at treatment initiation (years)**						
Median: >5.9863 vs. ≤5.9863	288	0.557 [0.34; 0.92]	0.0222			
**Height SDS at last visit**						
Median: >−2.4627 vs. ≤−2.4627	291	0.576 [0.35; 0.94]	0.0265	269	0.500 [0.29; 0.86]	0.0130
**Registry participation while receiving treatment (years)**						
Median: >4.9405 vs. ≤4.9405	266	1.567 [0.94; 2.62]	0.0871			
**Height velocity at treatment initiation (SDS/year)**						
Median: >−1.2089 vs. ≤−1.2089	149	1.033 [0.58; 1.84]	0.9116			
**Puberty onset at inclusion (yes/no)**						
Yes vs. no	291	0.565 [0.23; 1.41]	0.2221			
**Bone age at treatment initiation (years)**						
Median: >7 vs. ≤7	114	0.909 [0.42; 1.97]	0.8088			
**GH dose at inclusion (mg/kg/day)**						
Median: >0.0392 vs. ≤0.0392	273	1.331 [0.82; 2.16]	0.2471			
**GH dose at last visit (mg/kg/day)**						
Median: >0.0406 vs. ≤0.0406	288	0.808 [0.51; 1.27]	0.3591			
**IGF-I levels at inclusion (SDS)**			0.4857			
>+2 SDS vs. <−2 SDS	101	0.502 [0.10; 2.51]	0.4019			
–2 SDS to +2 SDS vs. <−2 SDS	101	0.434 [0.11; 1.71]	0.2330			
**Birth weight SDS**						
Median: >−1.7643 vs. ≤−1.7643	278	0.670 [0.41; 1.08]	0.1033			

Parameter	Univariate analysis			Multivariate analysis		
	n used	OR [95% CI]	P-value	n used	OR [95% CI]	P-value
**Cumulative dose during first 2 years of treatment (mg/kg)**			0.8495			
25.5675 ± 10% vs. >28.12425	266	1.175 [0.65; 2.12]	0.5899			
<23.01075 vs. >28.12425	266	1.124 [0.62; 2.05]	0.7017			
**Bone maturation at inclusion (years)**						
Median: >1.5106 vs. ≤1.5106	114	0.617 [0.27; 1.39]	0.2453			
**BMI at inclusion (kg/m²)**						
Median: >14.8739 vs. ≤14.8739	276	0.915 [0.57; 1.47]	0.7163			

A univariate analysis using a logistic regression model of each of the parameter listed above separately was performed. All prognostic factors that demonstrated associations with the outcome <20% were included in the multivariate model. For multivariate analyses, a backward stepwise selection was used to remove nonsignificant variables (p≥0.05). The selection variables were stopped when no more variables could be removed from the model. At the end of this selection, the final model was obtained.

AE, adverse event; BMI, body mass index; CI, confidence interval; GH, growth hormone; IGF-I, insulin-like growth factor-I; OR, odds ratio; SAE, serious adverse event; SDS, standard deviation score.
